# The Effect of Equipment-Based Pilates (Reformer) Exercises on Body Composition, Some Physical Parameters, and Body Blood Parameters of Medical Interns

**DOI:** 10.7759/cureus.24078

**Published:** 2022-04-12

**Authors:** Atakan Yilmaz, Mert Ozen, Rukiye Nar, Harun E Turkdogan

**Affiliations:** 1 Emergency Medicine, Pamukkale University, Denizli, TUR; 2 Biochemistry, Pamukkale university, Denizli, TUR; 3 Institute of Health Sciences, Pamukkale University, Denizli, TUR

**Keywords:** fitness, reformer, medical intern, exercise, equipment-based pilates, body composition

## Abstract

Background: Pilates is a type of exercise that exerts positive effects on body composition and general health. This study set out to investigate the effects of equipment-based Pilates (reformer) exercise on body composition, some physical parameters, and blood parameters of medical interns showing a tendency toward sedentary life.

Materials and methods: The experimental group (EG) comprising 22 healthy internship students in the medical faculty performed Pilates reformer exercises for nine weeks. The control group (CG) consisted of 18 students who did not engage in any exercise program. The baseline and final parameters of all the participants were measured.

Results: The mean age of the experimental group (EG) was 23.68±1.29 years, while that of the control group (CG) was 24.78±3.44 (*p*=0.089). A significant difference was evident between the performance pre-test and post-test scores of the EG (*p*<0.05). However, a significant positive difference was noted only between the waist pre-test and post-test results in the body composition measurements (*p*<0.05). A significant rise in HDL and fasting blood sugar levels and a decrease in insulin levels was observed in the post-exercise biochemical parameters measured in the EG (*p*=0.05). When the EG and CG were compared, a significant difference was found only in HDL cholesterol values in relation to the differences between the pre-test and the post-test groups (*p*=0.024).

Conclusion: The positive data from performance tests, especially with its HDL-increasing and insulin-lowering effects in the EG, implicate that Pilates reformer exercises can produce a favorable effect on the healthy living standards of medical interns.

## Introduction

Among the greatest challenges of our modern life are inactivity and a sedentary lifestyle, which lead to deterioration in body composition, physical measurements, and blood parameters [1,2]. Performing regular exercise and staying physically active facilitate daily life, improve balance and mobility, maintain mental focus, and improve overall health [3]. The World Health Organization (WHO) describes physical activity as any physical movement triggered by the skeletal muscles that call for energy expenditure [4]. Mounting empirical data document the impact of Pilates exercise, one of the physical activity methods, on body composition, stamina, general health, mental faculty, and sleep quality, highlighting its favorable effects [5-7]. Pilates might be a convenient physical activity option for university students who stay away from sports activities due to future employment concerns. The anxiety of studying for the Medical Specialization Examination or the Tıpta Uzmanlık Sınavı (TUS) adds to the increasingly heavy course load of medical school students, lack of sleep, and the tiring and busy schedule of being a medical intern. This situation forces medical interns to make some sacrifices in their social lives. Such a scenario causes them to lead a sedentary life by devoting all their time, except for the working time in the hospital, to study at the desk.

On one hand, university years are designated as a period of deterioration of health-related habits but, on the other hand, physical activity habits acquired during this period may be a precursor to healthy practices at later ages [8,9]. While physical activity improves health-related quality of life, sedentary behaviors adversely affect it [1,7]. Physical activity also reduces lipid peroxidation levels in both adults and elderly individuals by strengthening the antioxidant defense system. Elderly active individuals reportedly show similar antioxidant activity and lipid peroxidation levels to young sedentary participants by engaging in regular exercise in order to slow down the age-related deterioration process [10]. Moderate exercise and an active lifestyle not only help to inhibit oxidative stress, but also allow for primary and secondary prevention of neurodegenerative diseases, including cardiovascular disorders, type II diabetes, metabolic syndrome, and Alzheimer's disease [11].

Previous evidence garnered from clinical research suggests that physical activity strongly correlates with blood parameters, physical parameters, and body composition measurements. However, there remains a paucity of research investigating the impact of reformer exercises on blood parameters, physical parameters, and body composition measurements of medical interns who tend to lead a sedentary lifestyle. Against this background, this study seeks to explore the impact of equipment-based Pilates (reformer) exercises on body composition, some physical parameters, and body blood parameters of medical interns.

## Materials and methods

Study design

This prospective study was approved by Pamukkale University Clinical Research Ethics Committee (number 2020/05) and dated March 3, 2020. The study population consisted of medical interns who gave their informed consent to participate in the study, had not suffered an injury in the last six months, and did not have a history of chronic disease. These participants were recruited based on some pre-determined inclusion and exclusion criteria. The current study was performed in the reformer hall of the Sports Sciences Research and Application Center of Pamukkale University, Denizli, Turkey. This research was supported by a grant from Pamukkale University, Scientific Research Projects Fund (2020KRM010).

This study was conducted on a cohort of medical interns that was divided into two groups: the experimental group (EG) and the control group (CG). The EG received reformer training one hour per day, twice in a week, for nine weeks. In the first week, the performance tests to be carried out were demonstrated live to the whole study cohort for two days, and they performed these tests (Appendix). This practice was conducted to eliminate the effect of learning. At the end of the first week, performance pre-test values ​​and baseline blood samples ​​were noted down. Subsequently, the participants were randomized into two distinct groups through a simple computerized randomization procedure. The EG participants were informed about the reformer exercises they were expected to perform. From the second week onwards, eight-week reformer training sessions were initiated by a certified reformer trainer. In the meantime, the CG participants were asked not to engage in any exercise program. After eight weeks of reformer practice, the post-tests and final blood values ​​of all the participants were recorded.

We noted the baseline and final values of approximately 8 ml of venous blood samples drawn from the participating medical interns after at least 10-12 h of fasting. After the collected blood samples were allowed to clot for 30 minutes at room temperature and centrifuged at 3000 rpm for 10 minutes, their serum was separated. Total cholesterol (TC), triglyceride (TG), high-density lipoprotein (HDL), low-density lipoprotein (LDL), fasting glucose, and insulin values ​​from a portion of the separated serum were analyzed in the Cobas 702 biochemistry autoanalyzer (Roche Diagnostics, Mannheim, Germany). Homeostatic model assessment insulin resistance (HOMA-IR) value was calculated as HOMA-IR=fasting glucose (mg/dL) X fasting insulin (uIU/mL)/405. Besides, a part of the serum was stored at -800C until the parameters showing total oxidant levels (TOL) and total antioxidant levels (TAL) were studied. At the end of nine weeks, the baseline and final TOL and TAL parameters of both groups were examined on a Cobas 501 biochemistry autoanalyzer (Roche Diagnostics, Mannheim, Germany) by using commercial kits (Rel Assay Diagnostics, Şehitkamil, Turkey). The oxidative stress index (OSI) was calculated using the TOL/TAL ratio.

The recruited medical interns were asked to be in a state of fasting and thirst for at least three hours prior to body composition measurements. All the measurements and performance tests were carried out with the same materials by an expert in the field.

Identification of body compositions

All the body composition measurements of the participants were carried out in the fasting state in the morning hours. Basal metabolic rate (BMR), body mass index (BMI), body fat ratio (BFR), lean body mass (LBM), fat mass (FM), total body fluid ratio (TBFR), intracellular water (ICW), extracellular water (ECW) values ​​were all identified by means of a bioelectrical impedance analyzer, with all the participants in shorts and T-shirts [12]. They were informed not to consume alcohol, coffee, and other beverages affecting body water 24 hours before the measurements were made, and were also warned not to eat or drink anything at least three hours prior to the measurements.

Measurement of waist-hip ratios [13], sit-and-reach test [14], trunk-lateral flexion [15], sit-up test [16], sorensen test [17], active jumping test [18], were used as pre and post-tests.

Statistical analysis

The effect size reported in the reference study was observed to be strong (dz=0.67). Considering that a lower level of effect size (dz=0.6) could also be obtained, our power analysis calculated that 80% power could be obtained at the 95% confidence level, if at least 19 participants were included in the study. In addition, since a comparison with the CG, which was required not to do any exercise, was considered, a CG consisting of participants with the same number and characteristics as the EG was recruited (at least 38 people in total).

The collected data were analyzed with IBM SPSS Statistics for Windows, Version 25.0 (Released 2017; IBM Corp., Armonk, New York, United States). The continuous variables were presented as mean±standard deviation, median (minimum-maximum values), and categorical variables as numbers and percentages. A Shapiro-Wilk test was performed to see whether the data showed normal distribution. When the parametric test assumptions were met, a Student's t-test compared the independent group differences, and in the case of non-parametric test assumptions, a Mann-Whitney U-test was run to compare these differences. To compare the dependent group differences, a Paired Sample t-test was used in parametric test assumptions, and in non-parametric test assumptions, a Wilcoxon signed-rank test was carried out to calculate these differences. For all the statistical analyses, the significance level was set at p<0.05.

## Results

Of the initial cohort of 40 individuals who matched the selection criteria, there were 22 participants in the EG (reformer group) and 18 individuals in the CG. As far as their descriptive characteristics are concerned, the mean age was 23.68±1.29 years in the EG and 24.78±3.44 (p=0.089) in the CG. While the mean height turned out to be 172.18±7.01 cm in the EG and 170.61±10.62 cm in the CG (p=0.578), the mean weight was 65.29±13.08 kg in the EG and 67.18±12.36 kg in the CG (p=0.459).

As detailed in Table 1, the mean performance pre-test values of the EG were lower than those of the post-test. Moreover, a significant difference was evident between the jumping pre-test and post-test scores (p<0.05), whereas no significant difference was noted between the mean pre-test and post-test scores of the CG.

**Table 1:** Pre-test and post-test results of performance tests of experimental and control groups * The p-values ​​were obtained with Paired-Sample T test, and p <0.05 was considered significant.

	Experimental Group (N:22)							Control Group (N:18)								
	Pre-test	Post-test						Pre-test	Post-test							
	Mean ± Sd	Mean ± Sd	Δ (%)	p	t			Mean ± Sd	Mean ± Sd		Δ (%)		p		t	
Active Jump	24.27 ± 5.36	26.37 ± 6.23	8.65	0.0001*	-6.032		25.56 ± 9.4		25.66 ± 9.45	0.10		0.856*				-0.185
Sit-up Test	28.09 ± 7.81	35.41 ± 7.99	26.06	0.0001*	-7.344		35 ± 9.49		35.89 ± 9.24	0.89		0.196*				-1.344
Lateral Flexion	24.64 ± 4.84	27.73 ± 4.6	12.54	0.0001*	-5.53		24.17 ± 3.19		23.94 ± 2.71	-0.23		0.62*				0.506
Sit & Reach	36.05 ± 6.34	43.09 ± 6.08	19.53	0.0001*	-7.595		35.56 ± 7.66		36.39 ± 6.55	0.83		0.248*				-1.196
Sorensen	2.31 ± 0.73	2.65 ± 0.81	14.72	0.008*	-2.93		2.09 ± 0.72		1.86 ± 0.61	-0.23		0.044*				2.175

**Figure 1:**
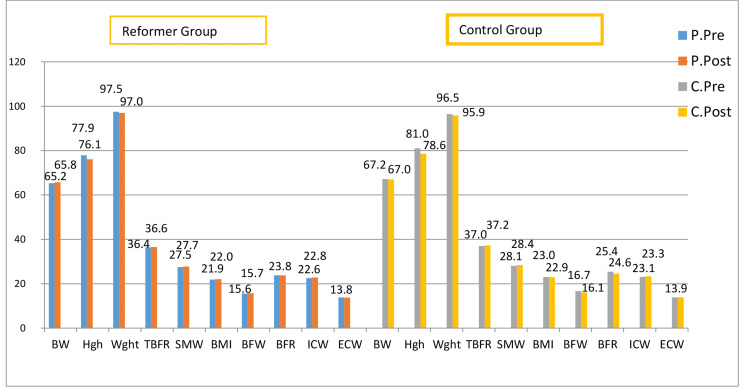
Pre-test and post-test results of body composition measurements of the EG and CG **BW: Body Weight; Hgh: Height; Wght: Weight; TBFR: Total Body Fluid Ratio; SMW: Skeletal Muscle Weight; BMI: Body Mass Index; BFW: Body Fat Weight; BFR: Body Fat Ratio; ICW: Intracellular Fluid; ECW: Extracellular Fluid; EG: Experimental Group; CG: Control Group; C.Pre: Control Group Pre-Test; C.Post: Control Group Post-Test; P.Pre: Pilates Pre-Test; P.Post: Pilates Post-Test

Figure 1 compares the mean of the pre-test and post-test values ​​of the body composition measurements of both groups. Accordingly, the EG did not indicate a significant difference between the pre-tests and post-tests with respect to BW, hips (H), TBFR, SMW, BMI, BMI, BFR, ICW, and ECW measurements. However, a significant positive difference was established only between the waist (W) pre-test and post-test results.

The measurements of the CG did not yield a significant difference between the pre-test and post-test values of BW, H, TBFR, SMW, BMI, and ECW. However, a significant negative difference was observed between the pre-test and post-test values of BFW, BMI, ICW, and W.

**Figure 2:**
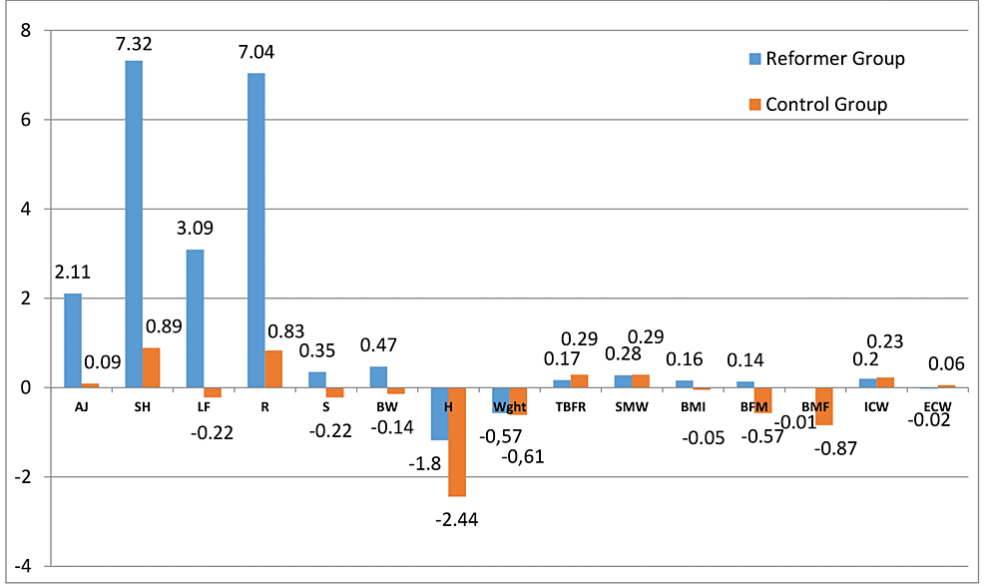
The difference between the performance and body composition measurement results of the EG and CG AJ: Active Jumping; SH: Shuttle; LF: Lateral Flexion; R: Reach Out; S: Sorensen; BW: Body Weight; Hgh: Height; Wght: Weight; TBFR: Total Body Fluid Ratio; SMW: Skeletal Muscle Weight; BMI: Height-Weight Ratio; BFW: Body Fat Weight; BMR: Body Fat Ratio; ICW: Intracellular Fluid; ECW: Extracellular Fluid

Figure 2 presents the results derived from the preliminary analysis of performance and body composition measurements of the EG and CG. Although a significant positive difference was noted between the performance tests of the EG and CG, no positive difference was evident in terms of body composition measurements. Besides, negative significant differences were found between W, BMI, and BFW measurements.

As listed clearly in Table 2, the baseline and final blood parameters of the EG, including TAL, TOL, OSI, TC, TG, LDL, and HOMA-IR did not reveal a significant difference, though a significant difference was obtained between HDL, FBG, and insulin values. In contrast, the CG did not show a significant difference between TAL, TOL, OSI, TC, HDL, FBG, Insulin, and HOMA-IR values, while their baseline and final TG and LDL levels differed significantly. Considering the differences between the pre-test and post-test in the blood parameters of the EG and CG, a significant difference was observed only in the HDL cholesterol value (p=0.024). Apart from that, all the other blood parameters did not differ significantly between the two groups in terms of differences in the decrease between the pre-test and post-test.

**Table 2:** Pre-test and post-test results of blood parameters in the EG and CG TAL: Total Antioxidant Level; TOL: Total Oxidant Level; OSI: Oxidative Stress Index; TC: Total Cholesterol; TG: Triglyceride; LDL: Low Density Lipoprotein;  HDL: High Density Lipoprotein; FBG: Fasting Blood Glucose; HOMA-IR: Homeostasis Model Assessment Insulin Resistance; EG: Ex[erimental Group; CG: Control Group *p values ​​were found by Paired-Sample T-test, and p <0.05 was considered significant. **p values ​​were found by Wilcoxon signed-rank test, and p<0.05 was considered significant.

	Experimental Group (N:22)			Control Group (N:18)		
	Pre-test	Post-test		Pre-test	Post-test	
	Mean ± Sd	Mean ± Sd	p	Mean ± Sd	Mean ± Sd	p
TAL	1.4 ± 0.17	1.41 ± 0.14	*0.67	1.4 ± 0.16	1.35 ± 0.12	*0.081
TOL	39.94 ± 17.61	34.02 ± 11.31	**0.408	33.34 ± 21.51	27.22 ± 8.6	*0.177
OSI	28.97 ± 13.61	24.06 ± 7.31	**0.355	23.59 ± 13.98	20.41 ± 7.49	*0.29
TC	159 ± 28.51	163.45 ± 32.01	*0.278	156.83 ± 30.13	151.44 ± 28.33	*0.058
TG	85.77 ± 27.37	83.45 ± 26.21	*0.679	76.61 ± 32.25	92.22 ± 47.91	**0.05
LDL	89.32 ± 27.12	90.5 ± 30.56	**0.794	86.56 ± 28.3	80 ± 23.94	*0.05
HDL	52.55 ± 13.41	56.23 ± 14.72	*0.05	55.06 ± 15.82	53 ± 15.39	*0.218
FBG	74.64 ± 7.3	79.77 ± 8.22	*0.022	82.11 ± 10.02	84.11 ± 7.71	*0.524
Insulin	7.69 ± 2.87	5.83 ± 3.06	*0.031	8.94 ± 4.07	10.43 ± 12.3	**0.215
HOMA-IR	1.44 ± 0.59	1.16 ± 0.65	*0.089	1.84 ± 0.98	2.23 ± 2.84	**0.309

## Discussion

As identified by many studies, an overwhelming majority of medical interns take the Medical Specialization Exam, hoping to practice as a specialist physician or academician in the later phase of their lives [5,6]. As a result, medical interns allocate almost all their time to studying at the desk, except for the working periods in the hospital, which leads to a sedentary life for these prospective professionals. There are many lines of evidence revealing that health-related quality of life is enhanced positively by physical activity but adversely affected by sedentary behaviors [1,2,7,19]. Although university years are characterized as a period of deterioration of health-related habits [20,21], physical activity habits developed during this period play a crucial role in turning them into health benefits in the following stages of their lives. A positive body image is also a significant contributor to individuals’ cognitive and psychological health [22]. Baek and Jeong report that individuals’ body image brings about favorable outcomes in their own psychological well-being, including physical and mental health, and all other activities concerning a healthy diet [23]. The results of a 16-week study conducted by Curi et al. implicate that stamina, aerobic capacity, flexibility, and dynamic balance capacity enhanced positively in the group performing Pilates exercises in comparison to the control group [6]. Our results obtained from stamina and flexibility tests are also validated against those reported by Curi et al. Moreover, a similar study performed on 33 participants highlights the contribution of Pilates exercises to the increase in muscular strength and flexibility [24], which is broadly supported by our findings. In another study by Suna and Isildak, which addresses the same subject, a significant difference was found between the group doing mat Pilates and reformer exercises and the control group in some performance test results (i.e. stamina, flexibility), as in our case [25]. However, the body composition measurements ​in the aforementioned study are not compatible with our findings. The primary reason for this seems to be the lack of nutritional intervention, one of the limitations of our study, and the secondary reason is the number of days of exercise. The reformer exercises were performed three days a week in Altıntaç’s study but twice a week in our study. There are other similar investigations in the literature reporting parallel results. In one of those investigations, Martins et al. evaluated the pre-test and post-test results of functional quality of life and body composition measurements of 78 participants (39 controls and 39 participants in the Pilates group who exercised twice a week for an hour a day for eight weeks) [26]. They established a positive increase in the functional life capacities of the Pilates group relative to the control group, while no significant difference was noted in their body composition measurements. Accordingly, their findings bear some marked similarities to those in our study. It is assumed that the main reason for a lack of difference in body composition measurements in this study, as in our study, may result from exercising only for one hour and two days a week. 

It is well established that physical activity triggers reactive oxygen radicals inducing cell damage. Although a fall in oxidative stress markers and a rise in antioxidants in our Pilates group were observed, no significant change was revealed. Lima et al. report that no increase in oxidative stress was evident in moderate-intensity exercise, while oxidative stress and muscle damage tended to increase in high-intensity exercise [27]. Whereas some research reveals that resistance exercise type did not enhance oxidative stress [28], some other studies suggest that exercises are likely to reduce the damage caused by oxidative stress [29]. Likewise, other clinical investigations document the role of endurance exercises in reducing oxidative stress and enhancing antioxidants [30]. An empirical study exploring different exercise models such as running, playing football, and skiing, reports a noticeable increase in both oxidant and antioxidant production [31]. Even low-intensity physical activities, such as fishing, walking, and team games, potentially reduce the damage induced by oxidative stress [32].

The present study found a significant rise in the baseline and final HDL cholesterol levels in the comparison of lipid profile examinations at the end of the eighth week, while no significant change was observed in other lipid profiles. Yasmin et al. report a significant improvement in the lipid profiles of the patients with type II diabetes at the end of a 12-week Pilates program [33]. A study on obese women that compares walking and Pilates exercises reveal that HDL and LDL changes tended to be lower in the Pilates group [34]. According to a study conducted in Iran that compared sedentary women and Pilates exercise groups, triglyceride changes were significantly different between the groups, yet no notable difference was found in HDL and LDL values [35]. A 12-week study comparing low-intensity cardio exercises vs. Pilates exercises in the Turkish context did not identify a remarkable change between baseline and final lipid values ​​in the latter group, while LDL and total cholesterol levels dropped significantly in the former group [36].

The final insulin resistance values did not differ significantly in the EG, but we detected a decrease in basal insulin levels and a rise in fasting blood sugar. Yanagawa et al. likewise observed no marked change in the insulin resistance of their participants after a 12-week exercise program [37]. In another research based on a 12-week schedule, a noticeable fall was noted in basal insulin and insulin resistance in the exercise group, while no significant difference was established in their fasting blood glucose level [36]. Previous literature reports suggest that lifestyle changes coupled with physical exercises produce favorable effects on insulin resistance [38,39].

The major limitation of our study is that medical interns develop irregular eating habits under heavy work and course load and that there was a lack of a nutrition plan that standarized their diet. A secondary limitation is that the volume of exercise, weekly lessons, as well as the number of days and hours might have remained insufficient.

## Conclusions

Our study sheds new light on the effects of reformer exercises on body composition, physical parameters, and body blood parameters of medical interns. Given the results derived from our performance tests, there is a marked increase in the sporting performance of medical interns. Our results bear striking similarities to the ones reported by other studies conducted on different groups, most notably in terms of performance tests. The changes in medical interns’ performance tests are suggestive of neural development. Furthermore, our findings obtained from the body composition measurements indicated no significant difference. Reformer exercises can help to maintain healthy living standards of medical interns by increasing their blood parameters, especially HDL, and lowering insulin. The present study is of great value in that it provides the first comprehensive assessment of the impact of reformer exercises on medical interns and proves useful in expanding our understanding of how these exercises affect their body composition, physical parameters, and body blood parameters. Further empirical evidence encompassing nutrition programs of medical interns is required in this field.

## Appendices

Pilates reformer exercises

(1) Pilates Footwork: Six Stance Positions

(2) Pelvic Lift

(3) Hundreds Prep Arm Circles: Drawing Down, Circles Flexion

(4) Hundreds

(5) Long Box Pulling Straps

(6) Long Box Seated Arms: Chest Expansion Seated, Biceps Curls, Serving Bread

(7) Scooter

(8) Reverse Scooter

(9) Eve's Lunge

(10) Repeat Pilates Footwork
